# Visuopathy of prematurity: is retinopathy just the tip of the iceberg?

**DOI:** 10.1038/s41390-021-01625-0

**Published:** 2021-06-24

**Authors:** Sigrid Hegna Ingvaldsen, Tora Sund Morken, Dordi Austeng, Olaf Dammann

**Affiliations:** 1 Department of Neuromedicine and Movement Science, Norwegian University of Science and Technology, Trondheim, Norway; 2 Department of Ophthalmology, St. Olav Hospital, Trondheim University Hospital, Trondheim, Norway; 3 Department of Public Health and Community Medicine, Tufts University School of Medicine, Boston, MA USA; 4 Department of Gynecology and Obstetrics, Hannover Medical School, Hannover, Germany

## Abstract

Research on retinopathy of prematurity (ROP) focuses mainly on the abnormal vascularization patterns that are directly visible for ophthalmologists. However, recent findings indicate that children born prematurely also exhibit changes in the retinal cellular architecture and along the dorsal visual stream, such as structural changes between and within cortical areas. Moreover, perinatal sustained systemic inflammation (SSI) is associated with an increased risk for ROP and the visual deficits that follow. In this paper, we propose that ROP might just be the tip of an iceberg we call visuopathy of prematurity (VOP). The VOP paradigm comprises abnormal vascularization of the retina, alterations in retinal cellular architecture, choroidal degeneration, and abnormalities in the visual pathway, including cortical areas. Furthermore, VOP itself might influence the developmental trajectories of cerebral structures and functions deemed responsible for visual processing, thereby explaining visual deficits among children born preterm.

## Impact

- This paper proposes that retinopathy of prematurity (ROP) is just the tip of the iceberg of an entity we call visuopathy of prematurity (VOP). VOP comprises the abnormal vascularization of the retina and visual processing abnormalities experienced by children born prematurely, even in the absence of ROP.
- This article contributes with a new perspective on the existing literature concerning visual abnormalities among children born prematurely.
- This article presents a new idea regarding the cause of visual abnormalities seen among children born premature, which will guide further research on the topic.

## Introduction

Retinopathy of prematurity (ROP) is a disease of abnormal neovascularization of the retina that occurs predominantly among very preterm infants. Four major risk factors are immaturity at birth (low gestational age (GA) and birth weight), exposure to fluctuating oxygen levels, perinatal/neonatal infection, and sustained systemic inflammation (SSI)^1,2^. Immaturity at birth is arguably one of ROP’s most critical risk factors, and the incidence of ROP increases with decreasing gestation and birth weight. In general, >50% of preterm infants weighing <1250 g at birth show evidence of ROP, and about 10% develop severe disease^3^. In our own previous work, we have explored the hypothesis that perinatal inflammation is associated with an increased risk for ROP^4–6^. We^7,8^ and others^9–11^ have provided empirical evidence in support of this concept, buttressed by experimental data^12–14^.

Most ROP research has focused on the abnormal retinal vascularization patterns that ophthalmologists can visualize by retinal examination. However, changes in retinal cellular architecture and relevant central pathways, including the primary visual cortex and extrastriate visual cortical areas, may also affect visual and cognitive function^15^. Some of these neurovascular changes may be explored using magnetic resonance imaging (MRI), electroretinography (ERG), and optical coherence tomography (OCT).

Already in 2006, some of us suggested to expand the concept of inflammation-associated brain damage in preterm infants to include visual dysfunction^16^. We now have evidence that ROP might not just be a vascular but a *neuro*vascular disease that involves both the retinal and cerebral neurovascular interphase^17^.

In this paper, we propose that ROP might be just the tip of the iceberg of an entity we have come to call *visuopathy of prematurity* (*VOP*). We postulate the existence of a spectrum of inflammation-associated changes to the retina (both cellular and vascular) and the brain’s visual pathways. These changes should not be considered separate from ROP but conceptualized as an overarching, inflammation-initiated entity that explains the spectrum of visual dysfunction in preterm infants better than ROP alone.

**Fig. 1:**
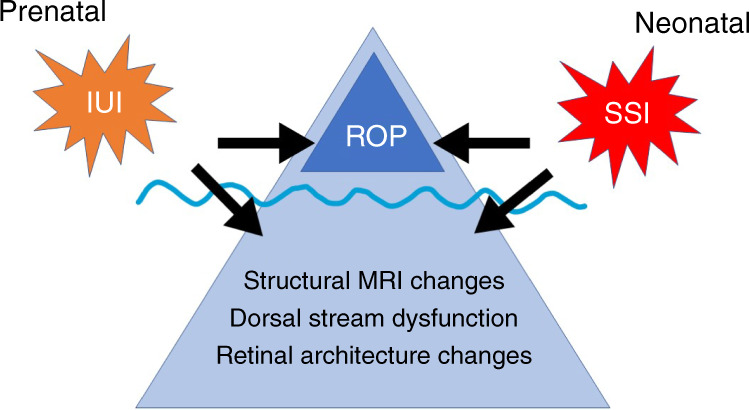
**ROP might just be the tip of the iceberg of a hypothesized entity called visuopathy of prematurity, comprising the vasculogenic components of ROP as well as structural MRI changes, dorsal stream dysfunction, and retinal architecture changes.** Prenatal and postnatal exposure to sustained systemic inflammation is a plausible etiologic factor for both the ROP and non-ROP components of VOP. ROP retinopathy of prematurity, MRI magnetic resonance imaging, SSI sustained systemic inflammation, IUI intrauterine inflammation.

We offer support for the VOP paradigm based on evidence from experimental work^18,19^, MRI^20,21^, ERG/electroencephalography (EEG)^15,22,23^, and OCT^24,25^. Taken together, the data support the notion that SSI in preterm newborns^26^ might contribute to a prolonged pathogenetic process that culminates in clinical VOP (Fig. 1) and explain the constellation of risk factors, imaging correlates, and clinical hallmarks of VOP. In what follows, we briefly review visual abnormalities seen among preterm infants with and without ROP, in addition to neurophysiological findings in these groups compared to full-term infants. Further, we describe how SSI might contribute to visual abnormalities beyond ROP. Finally, we will discuss our proposal based on vascular and neurovascular findings.

In sum, we propose that (1) VOP is an entity that includes the vasculogenic components of ROP as well as other retinal and cerebral abnormalities; (2) these visual abnormalities can occur among preterm infants even in the absence of ROP; and (3) prenatal and postnatal exposure to SSI is a plausible common etiological contributor to the ROP and non-ROP components of VOP. We hope that our proposal may help develop further research into the origins of visual abnormalities in children born preterm.

## OCT of the preterm retina

OCT imaging of the macula area is a reliable and useful tool to identify pathologic changes in the retina’s microstructure due to its non-invasive, real-time cross-sectional imaging of live tissue^27^. Retinal and optic nerve head abnormalities seen on OCT images may be biomarkers for VOP. For example, macular edema is associated with later neurodevelopment and cerebral visual impairment in preterm infants^28,29^, the optic nerve head rim area is smaller in preterm infants than full-term peers at school-age^30^, and increased retinal thickness is associated with decreased visual acuity^31^.

High-resolution spectral-domain OCT demonstrates a reduced thickness of the retinal nerve fiber layer (RNFL) in very preterm infants, which is associated with abnormal visual function and white matter injury at term-equivalent age^24^. The RNFL consists of the unmyelinated ganglion cell axons that form the optic nerve, which extends as the optic tract into the lateral geniculate nucleus (LGN) of the thalamus. Reduced RNFL thickness is associated with more extensive brain abnormalities on MRI^25^, pointing toward OCT findings as a correlate and perhaps even biomarker for cerebral abnormalities in preterm infants. Findings regarding the role of RNFL thickness in the context of ROP are inconsistent. One study attributed reduced RNFL thickness at lower GA to ROP^32^. In contrast, another study found no such association^25^. Instead, RNFL thickness was associated with birth weight, GA, and MRI abnormality, suggesting that reduced RNFL thickness is a correlate of retinal immaturity rather than ROP.

Preterm infants also display increased foveal thickness compared to children born full term^33,34^, and this phenomenon is even more prominent among preterm infants with ROP^31,35,36^. In a multiple regression analysis including GA, birth weight, neurological complications, refraction, visual acuity, ROP, and age at examination, GA was the only significant risk factor for a thicker macula among preterm infants^37^. However, any inference from multivariable modeling based on statistical significance should always be made with much caution because confounder constellations may change during the modeling process of adding or eliminating co-variables.

Inner and outer cell layer thickness is also strongly correlated with GA, and the stage of ROP has no impact on the degree of foveal development^33,34^. The foveal photoreceptor layer is significantly thinner among infants with GA ≤28 weeks compared to full-term infants, independent of ROP manifestation^31^. This suggests that the degree of immaturity indicated by GA, as well as its antecedents, correlates, and consequences, might be better indicators of abnormal retinal development than ROP alone.

## Electrophysiological studies

Essential steps for retinal development occur between post-conceptual weeks 25 and 38. During this period, photoreceptors develop distinct inner and outer segments, the avascular zone in the fovea is created, and the outer plexiform layer and the retinal vasculature extend to the retinal periphery^38^. The ERG responses of the immature retina reflect the immaturity of photoreceptors. The ERG a-wave measures the response from photoreceptors (rods and cones), while the b-wave primarily reflects the bipolar cells’ response. Children born preterm display lower amplitudes of the combined rod and cone responses, in addition to the peak of the isolated cone response compared to full-term infants^38,39^, which suggests a general retinal dysfunction of both the photoreceptors and the cone bipolar cells.

An ERG study of school-age children revealed that those born before the 27th week of gestation had a significantly prolonged cone-driven 30-Hz flicker ERG implicit time compared to children born at term. However, the 30-Hz flicker ERG amplitudes showed no significant differences between preterm infants with and without ROP^22^, which suggests reduced retinal function due to retinal immaturity without an additional effect of ROP. Multifocal (mf)^40^ and full-field (ff) ERG studies^41^ have yielded similar results. The amplitude of mfERG/ffERG responses in children born preterm is significantly reduced compared to their full-term peers, even if they never had ROP. This suggests that photoreceptor function is reduced in children born preterm and that these effects on the neurosensory retina do not appear to depend on the vascular and angiogenetic characteristics of ROP^40,41^. However, in children born preterm who had ROP, a prolonged rod photoreceptor response deficit is detectable after the active ROP disease has resolved. The increased receptive field size seen in these children may compensate for the deficient receptor input to the post-receptor retinal neurons^42^.

Electrophysiological studies have also revealed photoreceptor abnormalities among preterm infants. Visual evoked potentials (VEPs) measure the latency of the visual signal transmission from the retina to the occipital cortex^43^. One VEP study found that >25% of premature infants deemed to be at low risk for abnormal development display a slow response to motion stimuli at 3 months, consistent with visual attention and cortical processing deficits^15^. Moreover, this slow response is present in the visual pathways of preterm infants without appreciable retinal or neurologic impairment^43^. This notion is supported by a study that compared visual acuity, refraction, optic disc parameters, and VEP in moderate-to-late preterm (MLP) infants (GA between 32 and 36 weeks) to similar measures obtained from infants born at term^44^. Investigators saw no significant differences in the retinal measures, but VEP amplitudes were lower in the MLP infant group. Furthermore, very low birth weight infants exhibit significant changes in cortical responsiveness in the absence of morbidity, such as ROP or cerebral white matter abnormalities^44,45^, indicating an impairment of central visual processing development.

## Development of central visual processing and its impairment in preterm infants

Central visual processing entails sensation, visual perception, visual and spatial cognition, and visuomotor processing. Even though these functions are not associated with a clear division in the interacting brain network, visual developmental pathologies in the preterm brain are related to visual functioning in different eye–brain networks^46^. After an image has been formed in the retina and photoreceptors encode the image as neural signals, they are transmitted through the optic nerve and optic radiation. The majority form connections in the primary visual cortex and further transmit information through extrastriate visual areas, forming two distinct streams, the dorsal stream and the ventral stream^47,48^. Subtle visual deficits among children and adults born preterm are associated with abnormal dorsal stream function, even in the absence of major neurological disabilities^49,50^. These visual functions include motion processing, understanding spatial relations, and visuomotor control^51^. In addition to this dorsal stream vulnerability, preterm infants are at increased risk of cerebral white and gray matter damage due to infection and subsequent neuroinflammation^52^, which may affect any component of the visual pathway from the retina to the visual cortex.

Cerebral visual impairment is the impairment of visual acuity due to damage to visual cortical areas and accounts for nearly 30% of all bilateral visual impairments among young children^53^. The visual system is in the close vicinity of the white matter adjacent to the cerebral ventricles, a predilection site for brain abnormalities in preterm infants. Abnormalities in this paraventricular area occur more frequently toward the posterior parietal lobe and involve optic radiations and nearby corticospinal tracts. These white matter abnormalities seen among preterm infants are associated with neuronal and axonal disease in the brainstem, basal ganglia, thalamus, cerebral cortex, and cerebellum^15,54^. Cerebral visual impairment is also associated with lesions posterior to the optic chiasma, which may be related to ROP^15,17,55^. Moreover, children with ROP are more likely than their peers to have cognitive and psychomotor development below the expected mean at 2 years of age^21^.

Significant differences in white matter density exist between children born preterm and their full-term peers^20,21,56,57^. Diffusion tensor imaging studies have revealed significant differences in several white matter regions between preterm and full-term infants, but only a few differences between preterm infants with and without ROP^20^. However, structural network analyses have revealed a significant difference in brain connectivity between those with and without severe ROP, indicating that while preterm birth may be more strongly associated with white matter maturation than ROP, severe ROP may still be associated with decreased structural connectivity. Moreover, compared to full-term infants, there are remarkable differences in the degree of diffusion between regions and overall mean diffusivity values in preterm infants regardless of ROP^20^. This suggests that delayed maturation of the dorsal and ventral pathways in infants with ROP might be attributed to immaturity rather than ROP alone.

This notion is further supported by studies examining global visual processing in the dorsal stream. Preterm infants display difficulties with global visual processing attributed to the dorsal stream^50,51^. The dorsal stream, connected with the parietal cortex, has a more protracted developmental course than the ventral stream, which is connected with the temporal cortex, and could thereby provide an extensive opportunity for atypical exposure to affect cortical areas’ function in this system. Brain regions within the dorsal stream are particularly vulnerable to the diffuse white matter injury among preterm infants^50,54,58^. Some preterm infants without ROP have white matter abnormalities associated with dorsal stream dysfunction^59^, pointing to the parietal lobe as a core cortical region for abnormal visual processing in preterm infants. Moreover, the degree of immaturity at birth appears to be essential in determining the degree of visual global processing deficits later on^50^.

These visual processing abnormalities among preterm infants could be explained by the fact that both the retina and brain regions in the dorsal stream may be vulnerable to the insufficient availability of trophic factors. For example, insulin-like growth factor 1 (IGF-1) is critical for both vascular and neural development, at least in part by modifying the systemic levels of vascular endothelial growth factor (VEGF), which plays a crucial role in the pathogenesis of ROP^60^, and also for the augmentation and utilization of glucose across neural cells in the brain^56^. The insufficient availability of IGF-1 may be associated with increased levels of inflammatory cytokines produced in the context of prenatal and sustained postnatal inflammation^61^. For example, increasing IL-6 levels are associated with decreasing IGF-1 levels during the first months after preterm birth, and infants treated for ROP have continuously higher postnatal levels of IL-6 than infants with no history of ROP, indicating a negative correlation between IGF-1 and pro-inflammatory cytokines among preterm infants, regardless of ROP^62^.

Another area of concern is the current strategy to treat ROP with intravitreally injected anti-VEGF agents due to its potential effects on the preterm brain^63,64^. Recent clinical reports have indicated that some anti-VEGF agents can leak from the eye into the systemic circulation in ROP infants and consequently inhibit neurodevelopment^65^. VEGF is an essential developmental molecule, and therefore VEGF inhibition may have long-term effects on central nervous system development, including visual processing. Extremely preterm infants at 18 months treated with anti-VEGF injections have lower motor scores and higher rates of severe neurodevelopmental disabilities than preterm infants treated with laser ablation^63^. Moreover, an experimental study has demonstrated that even a single injection of intravitreal anti-VEGF in mice with ROP inhibits their weight gain, suggesting that it can impair the systemic development of infants with ROP.^66^. The blood–brain barrier and blood–retina barrier may prevent anti-VEGF agents from spilling over into extravascular space, reducing the impact on brain vessels. Therefore, anti-VEGF leakage may have a more considerable impact on preterm infants with severe zone 1 ROP because they have a more immature avascular retina^65^.

## Prenatal and sustained postnatal inflammation

A rapid systemic inflammatory response is an effective defense mechanism against microbial invasion that usually resolves after the infection is cleared. However, sustained and dysregulated inflammation may damage organs and contribute to chronic autoimmune diseases in adults when it fails to resolve.

Among preterm infants, inflammatory responses are common before and after birth^67,68^ and are likely to be involved in ROP etiology and visual abnormalities among preterm infants^5,16,69,70^. These inflammatory responses may be a result of oxidative stress, and vice versa. Oxidative stress is the consequence of an imbalance between pro-oxidants and antioxidants. Antioxidants (including enzymes, vitamins, and minerals) protect the cells against the harmful effect of oxidants (free radicals). Preterm infants are more prone to oxidative stress because they are often exposed to high oxygen, infection, or inflammation and also have limited antioxidant defenses. Several pathological consequences of immaturity at birth associated with visual processing, such as ROP and white matter abnormalities, appear to be related to oxidative stress. Moreover, oxidative stress and inflammation are closely related in the form of a vicious circle. For instance, an inflammatory microglial response in cerebral white matter can generate free radicals, which in turn may induce pro-inflammatory cytokine production^71,72^.

Furthermore, inflammation and oxidative stress are closely intertwined stress responses implicated in the pathogenesis of retinal diseases, including ROP^73^. For example, both can lead to stress-induced premature senescence (SIPS), the premature loss of cellular replication capability that is a hallmark of the natural aging process^74^. At the same time, increased production of pro-inflammatory cytokine interleukin (IL)-6 and chemokine IL-8 is a key characteristic of oxidative stress-induced SIPS in retinal pigment epithelial cells^75^. We speculate that SIPS in the retina and/or elsewhere in the central visual system may be among the pathogenetic mechanisms contributing to long-term visual dysfunction in individuals born preterm.

Recent findings suggest that exposure to the pro-inflammatory cytokine IL-1β is associated with choroidal degeneration in the early stages of retinopathy and that fetuses exposed only to IL-1β display a persistent postnatal infiltration of inflammatory cells^18^. This prolonged inflammatory response is associated with (1) a substantial delay in retinal vessel growth, (2) long-lasting thinning of the choroid, and (3) long-term morphological and functional alterations of the retina, including outer retinal dysfunction (such as defective dark adaptions due to rod dysfunction, abnormal cone function, and retinal depigmentation)^18^. Because preterm infants with ROP often have higher systemic concentrations of inflammatory cytokines that may affect the choroid (a source of oxygen and nutrients to both the subretinal and outer retina), ROP should no longer be considered only a vasculopathy of the inner retina^19^. We are aware that any potential discrepancies between systemic and tissue levels of mediators of inflammation might be perceived as weakening our argument. On the other hand, evidence continues to accumulate in support of the notion that systemic inflammation does contribute to tissue abnormalities in the brain^26^ and eye^6^.

Preterm infants are predisposed to sepsis and other conditions that can contribute to the onset of SSI. Elevated concentrations of inflammatory mediators (such as tumor necrosis factor (TNF)-α, IL-1β, and IL-6) during the first postnatal month predict the occurrence of ROP^61^. Pro-inflammatory cytokines are overexpressed and insufficiently balanced by immunosuppressive elements, causing neuroinflammation and visual function deficits through three main cell types, i.e., astrocytes, microglia, and immune cells^76^.

Glia cells such as astrocytes are present in large parts of the retina during development and produce vasculogenic growth factors^77^. The vasculogenic process facilitated by growth factors such as the VEGF produced by astrocytes and retinal ganglion cells in the low oxygen environment in utero results in retinal neovascular abnormalities^4,78^. While VEGF is essential for physiological retinal angiogenesis, elevated VEGF in ROP contributes to pathological angiogenesis^79^. Retinal microglia can negatively affect the retina by producing IL-1β and TNF-α in response to hypoxia. In mice with oxygen-induced ROP, IL-1β is indirectly related to both the retina’s microvascular degeneration and the involution of choroidal vessels, resulting in subretinal hypoxia and disturbance of photoreceptor cell integrity^19^.

Microglia helps establish and mature neuronal circuitry during development, maintains neuronal cells, and mediates changes in synaptic circuitry and activity by regulating synapse and receptor expression^80^. In the retina, microglial cells remodel retinal vasculature during development by clearing excessive vessels and participating in vessel anastomosis. However, excessive microglial activation is associated with hypomyelination^80^. Severe systemic perinatal inflammation provokes microglia activation within the retina, leading to aberrant vascular development with excessive anastomosis, associated with long-term deficits in visual function^14^. For example, microglia are a source for IL-1β, and activated microglial cells in the hypoxic neonatal retina produce increased TNF-α and IL-1β, which may induce retinal ganglion cell layer death and consequently affect the visual capabilities of the child^81^. Furthermore, microglial cells respond to signals from the peripheral immune system inducing neuroinflammation and releasing proinflammatory cytokines. One of these cytokines, Gro1, triggers premature senescence in the newly developing neurons, which may lead to irreversible growth arrest of this cell population^82,83^. We therefore consider it likely that glia cells, and microglia cells in particular, play an important role in VOP pathogenesis.

## Conclusion: VOP as the Iceberg

The empirical and experimental studies discussed above provide evidence that neurovascular visual abnormalities and visual processing deficits are frequent among preterm infants, even in the absence of ROP^22,37,59^. However, abnormalities identified by ERG/EEG, OCT, and MRI seem to be more frequent and/or prominent among preterm infants with ROP^21,32,42^. What we call *visuopathy of prematurity* is a pathological entity among preterm infants that includes ROP, abnormal photoreceptor function, and subretinal abnormalities such as choroidal thinning, as well as abnormalities further down the visual tract. Retinal abnormalities in preterm infants with and without ROP revealed by ERG and OCT studies are associated with cerebral and visual acuity dysfunction^29,44^, suggesting that ERG and OCT findings might be biomarkers not only for ROP but also for VOP in general.

Both ROP and non-ROP components of VOP are associated with SSI, which may explain why some of the retinal and visual processing abnormalities of VOP also exist in preterm infants without ROP. We consider it likely that prenatal and sustained postnatal systemic inflammation is a core contributor to the neurovascular visual abnormalities, causing both retinal and cerebral dysfunctions. The exact mechanisms of how inflammation contributes to VOP remain to be elucidated.

In sum, we propose that (1) VOP is an entity that includes the vasculogenic components of ROP as well as other retinal and cerebral abnormalities, (2) these visual abnormalities can occur among preterm infants even in the absence of ROP, and (3) prenatal and postnatal exposure to SSI is a plausible common etiological denominator for the ROP and non-ROP components of VOP. We hope that our proposal will contribute to further research into the origins of visual abnormalities in children born preterm, thereby facilitating the design of therapeutic and preventive interventions.
